# The Vanishing Tumor: Parotid Oncocytoma

**DOI:** 10.7759/cureus.91115

**Published:** 2025-08-27

**Authors:** Sriya Srujana Paladugu, Abirami Raghunath, Chandru Ravindrakumar, Narayanan Cunnigaiper

**Affiliations:** 1 General Surgery, Sri Ramachandra Institute of Higher Education and Research, Chennai, IND

**Keywords:** benign tumor, fine needle aspiration cytology, histological examination, magnetic resonance imaging, parotid gland oncocytoma, superficial parotidectomy

## Abstract

Here, we report a case of a 58-year-old male who presented with a history of right parotid swelling with pain for more than a year. Initially, the swelling was diagnosed as an abscess for which the patient was treated with antibiotics followed by incision and drainage at an outside facility; however, the patient presented with recurrence. Magnetic resonance imaging (MRI) showed multiple hypointense lesions, suggesting the possibility of an oncocytic tumor. While fine needle aspiration cytology (FNAC) was nondiagnostic for malignancy due to an inflammatory background, it had not ruled out malignancy. He underwent a right superficial parotidectomy, along with drainage of purulent material suggestive of an abscess. Histopathological examination showed multifocal oncocytic hyperplasia and adjacent abscess formation; the diagnosis of oncocytoma was made. This case exemplifies the difficulty of diagnosing parotid oncocytomas and highlights how histological analysis can make such a diagnosis possible.

## Introduction

Parotid gland oncocytomas are rare, accounting for less than 1.5% of all parotid tumors. These benign neoplasms typically arise in the salivary glands [1]. The therapeutic management of oncocytomas is complicated by their histological similarity to other benign parotid tumors, such as pleomorphic adenomas and Warthin tumors. Oncocytomas can be histologically differentiated from other benign cystic tumors owing to the presence of oncocytic cells containing hyperplastic mitochondria [2]. They grow gradually over time, often manifesting as firm, painless masses that are rarely invasive, resulting in delayed diagnosis. This is a case of a patient presenting with a swelling in the right parotid region that persisted for over a year. The patient underwent multiple conservative treatments that included courses of oral antibiotics and, eventually, an incision and drainage before ultimately being diagnosed with a parotid oncocytoma and subsequently undergoing definitive management via superficial parotidectomy.

Oncocytomas predominantly affect older adults, typically presenting in individuals in their sixth to eighth decades of life, although they may occur earlier. The cellular composition and clinical features complicate the diagnosis of oncocytomas that are primarily benign. Imaging modalities, such as magnetic resonance imaging (MRI) and ultrasound, can aid the initial evaluation; however, a definitive diagnosis necessitates histological analysis following excision [3]. This case illustrates the diagnostic difficulty of parotid oncocytomas and the indispensable role of histopathology.

Parotid oncocytomas may recur, and their treatment regimens are extensive, necessitating differentiation from other parotid tumors [4]. On imaging, oncocytomas typically present as well-defined, lobulated lesions that exhibit intense enhancement on contrast imaging, indicative of their unique cellular structure with a high mitochondrial content [5]. Nevertheless, the radiographic characteristics that may lead to the misdiagnosis of an oncocytoma - overlapping with those of other parotid lesions - remain unchanged [6].

This report explores the clinical presentation, diagnostic process, and surgical management of a patient with a long-standing parotid oncocytoma, initially obscured by a recurrent parotid abscess and repeatedly treated under that assumption. The patient’s history of conservative treatments for parotid swelling and abscess discharge, along with imaging results indicating multiple T1 hypointense lesions and necrotic regions within the gland, demonstrates the tenacity of the oncocytoma. Histopathological investigation subsequently confirmed the diagnosis and justified the decision to perform a superficial parotidectomy; histopathology revealed oncocytic nodules with necrotic regions. The surgical outcome was favorable, with no recurrences or postoperative complications.

This case underscores the necessity for heightened clinical awareness regarding persistent parotid masses in patients exhibiting recurrent symptoms following conservative therapy. Considering the infrequent incidence and resultant diagnostic uncertainty associated with parotid oncocytomas, a multidisciplinary approach encompassing imaging, histology, and possibly surgical intervention is essential for preventing recurrence and optimizing patient outcomes [7]. This introduction, therefore, highlights the significance of early diagnosis and the requirement for histological confirmation in the broader context of managing parotid oncocytomas [8].

## Case presentation

A 58-year-old man presented with a history of right parotid swelling lasting approximately one year. The right parotid swelling developed insidiously, exhibited gradual progression, and was accompanied by intermittent fever and occasional pain. Notably, the patient had previously consulted another clinic for episodes of similar swelling, treated with antibiotics, an incision, and a drainage procedure. Nonetheless, the swelling recurred, prompting the patient to seek further evaluation at our facility. The patient reported no significant weight or appetite loss. The patient had no significant history of habits, and their family history was noncontributory. On examination, the patient exhibited right parotid swelling measuring approximately 4 × 5 cm. The overlying skin presented no erythema, localized temperature elevation, or ulceration. The mass had a smooth surface and was firm in consistency, with no regional lymphadenopathy. There were no significant intraoral findings.

**Figure 1:**
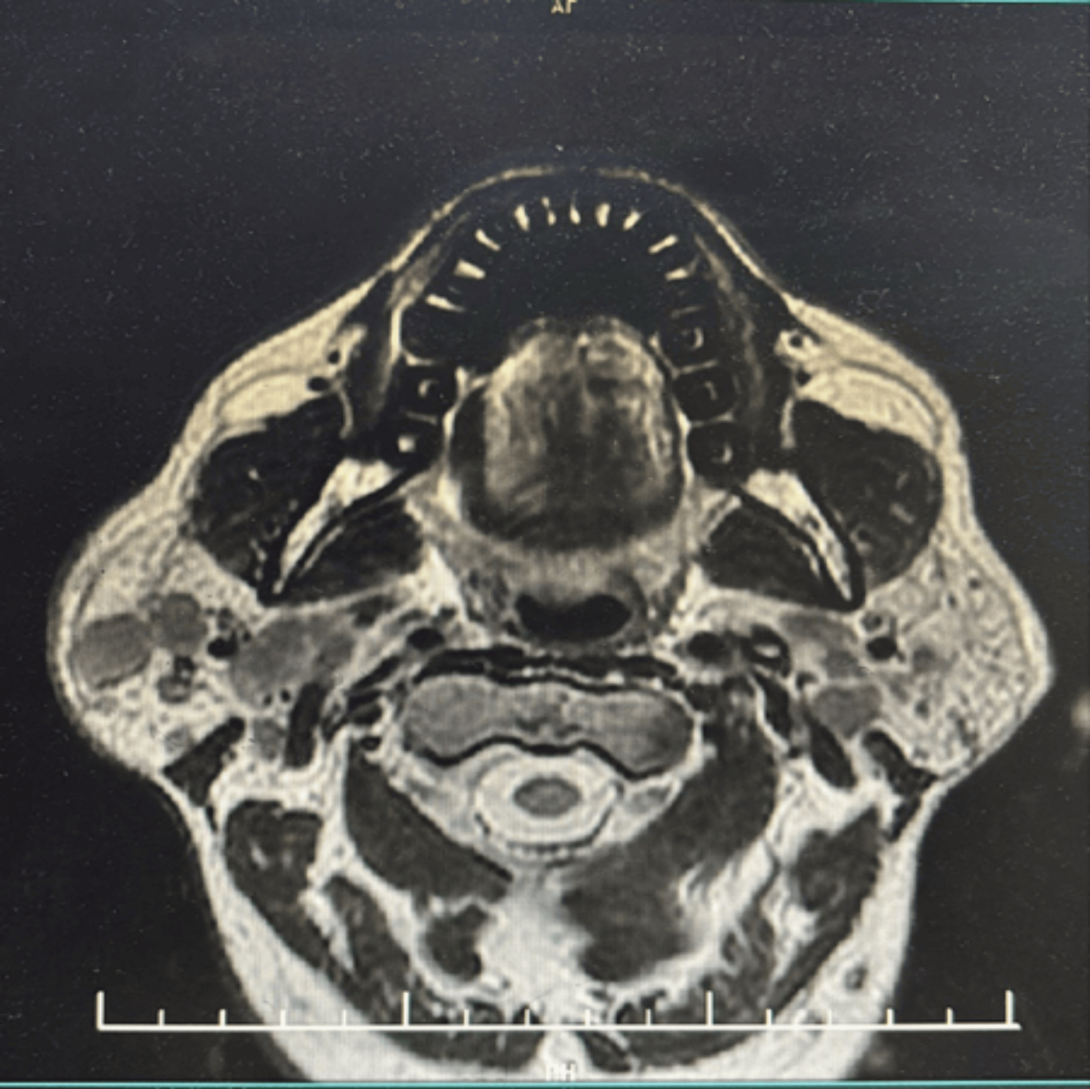
Axial T2WI of the patient The axial T2-weighted image (T2WI) shows multiple T2 hypo- to isointense lesions in the right parotid gland.

The initial magnetic resonance sialogram revealed multiple T1 hypointense lesions in the right parotid gland. The largest lesion in the inferior part of the right parotid gland measured approximately 16 × 17 mm. A third distinct lesion in the superior lobe of the right parotid measured 2 × 1.7 cm, exhibiting peripheral limited diffusion suggestive of secondary infection. However, the lesions were isointense to the native parotid gland on fat-saturated T2-weighted and post-contrast T1-weighted sequences, yielding the characteristic “vanishing tumor” appearance on MRI (Figure 1).

**Figure 2:**
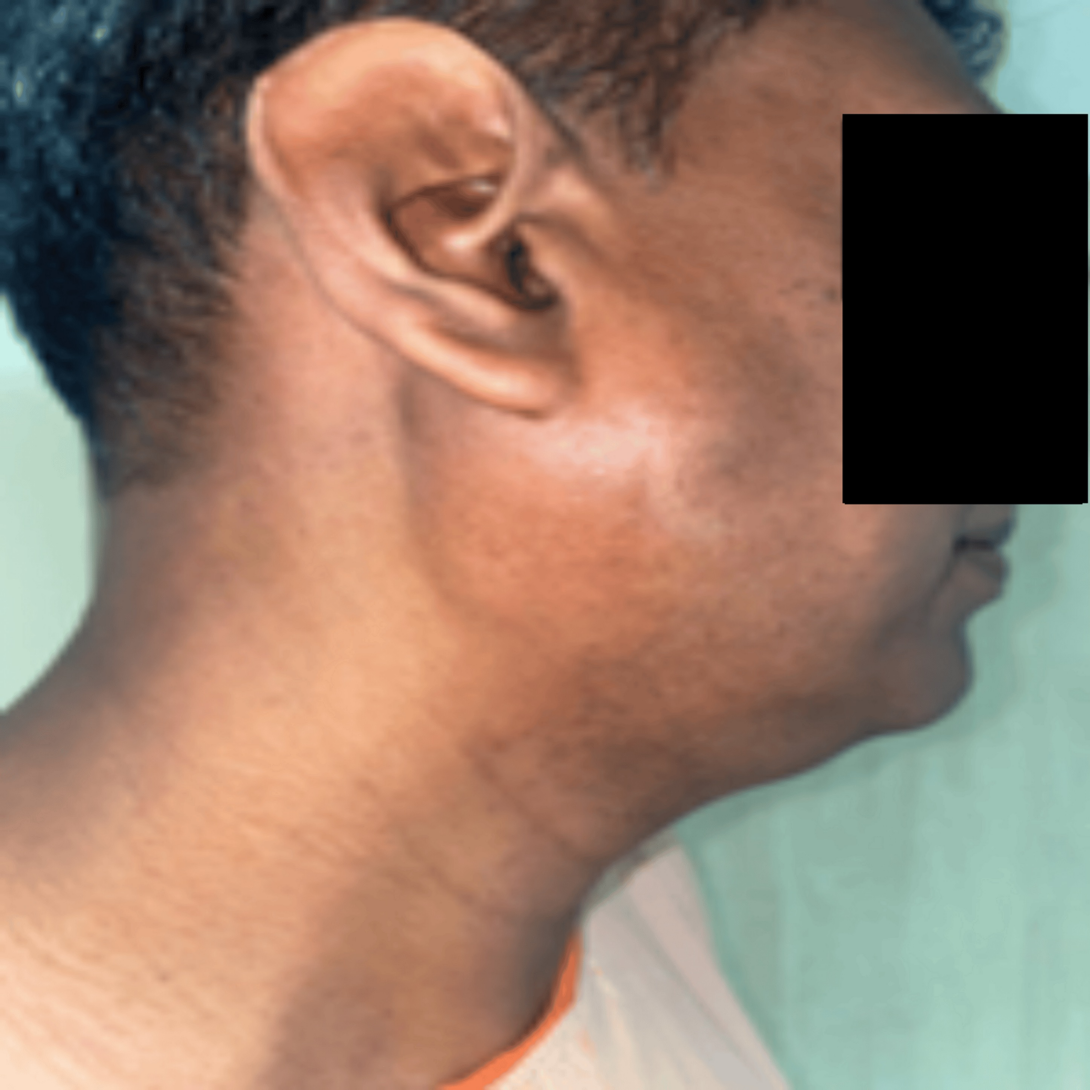
Preoperative image of the patient revealing right parotid swelling

Figure 2 depicts right parotid enlargement in the preoperative state. Histopathological examination correlation was recommended, considering the potential diagnosis of an oncocytic tumor or lymphadenopathy.

Fine needle aspiration cytology (FNAC) of the right parotid gland was performed. Microscopic examination of the specimen revealed a necrotic background with sheets of neutrophils, lymphocytes, and histiocytes, without any evidence of malignant cells.

The findings suggested a benign inflammatory process. Considering the imaging results and recurrent nature of the swelling, a right superficial parotidectomy was performed under general anesthesia. During the procedure, approximately 10 mL of purulent fluid was also drained from the affected area. The excised specimen was submitted for histological analysis.

**Figure 3:**
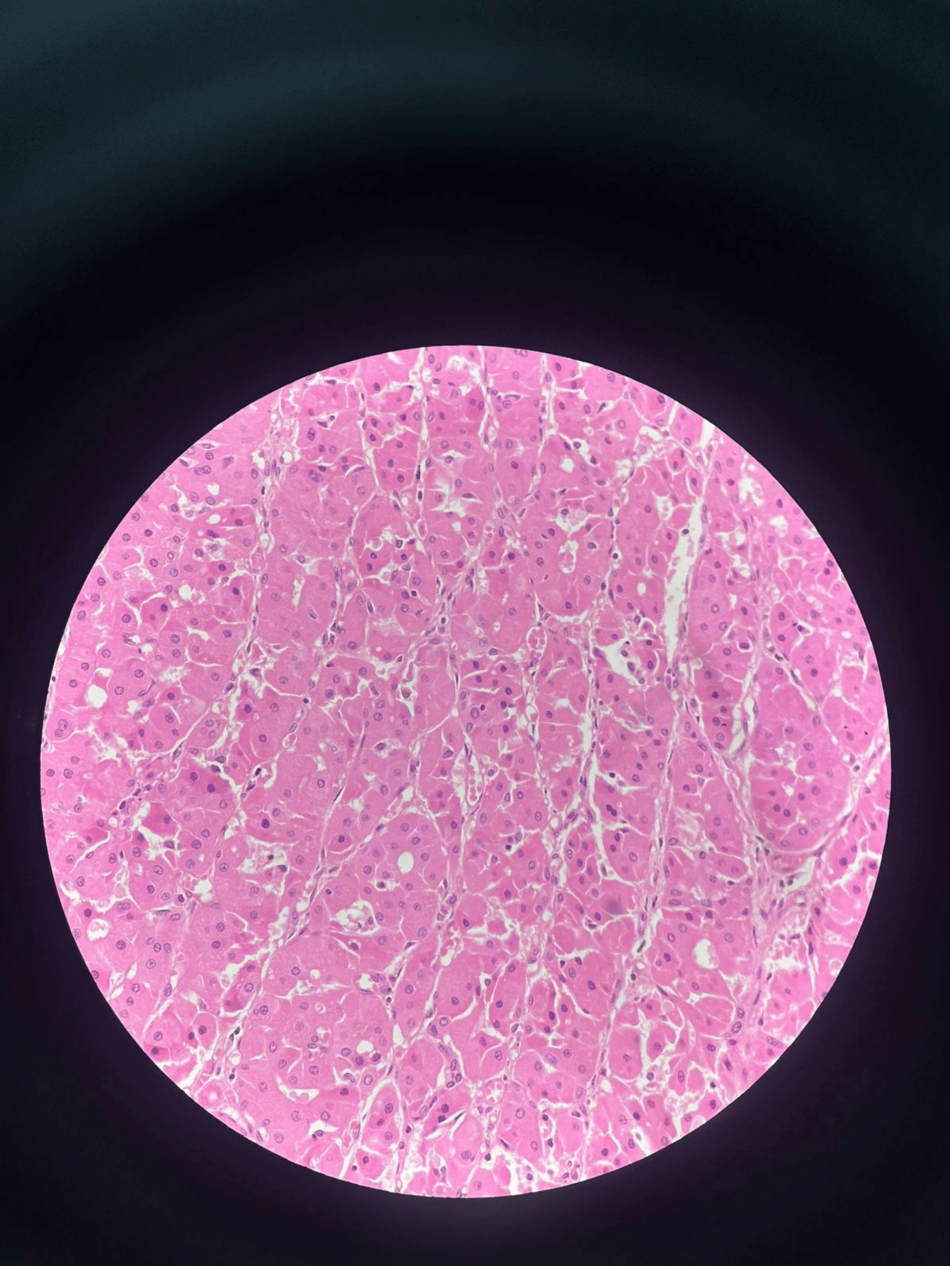
HPR analysis revealing small, round nuclei characteristic of oncocytic and large polygonal cells with eosinophilic granular cytoplasm HPR: Histopathological report.

Histological examination of the superficial parotidectomy specimen revealed a well-defined, encapsulated lesion comprising small, round nuclei characteristic of oncocytic cells, along with large polygonal cells exhibiting eosinophilic granular cytoplasm. Numerous nodular areas of oncocytic hyperplasia extended to the resected gland margins, alongside a significant inflammatory infiltrate primarily composed of neutrophils. The presence of adjacent abscess formation as well as multifocal oncocytic nodular hyperplasia and oncocytomas validated these findings (Figure 3).

**Figure 4:**
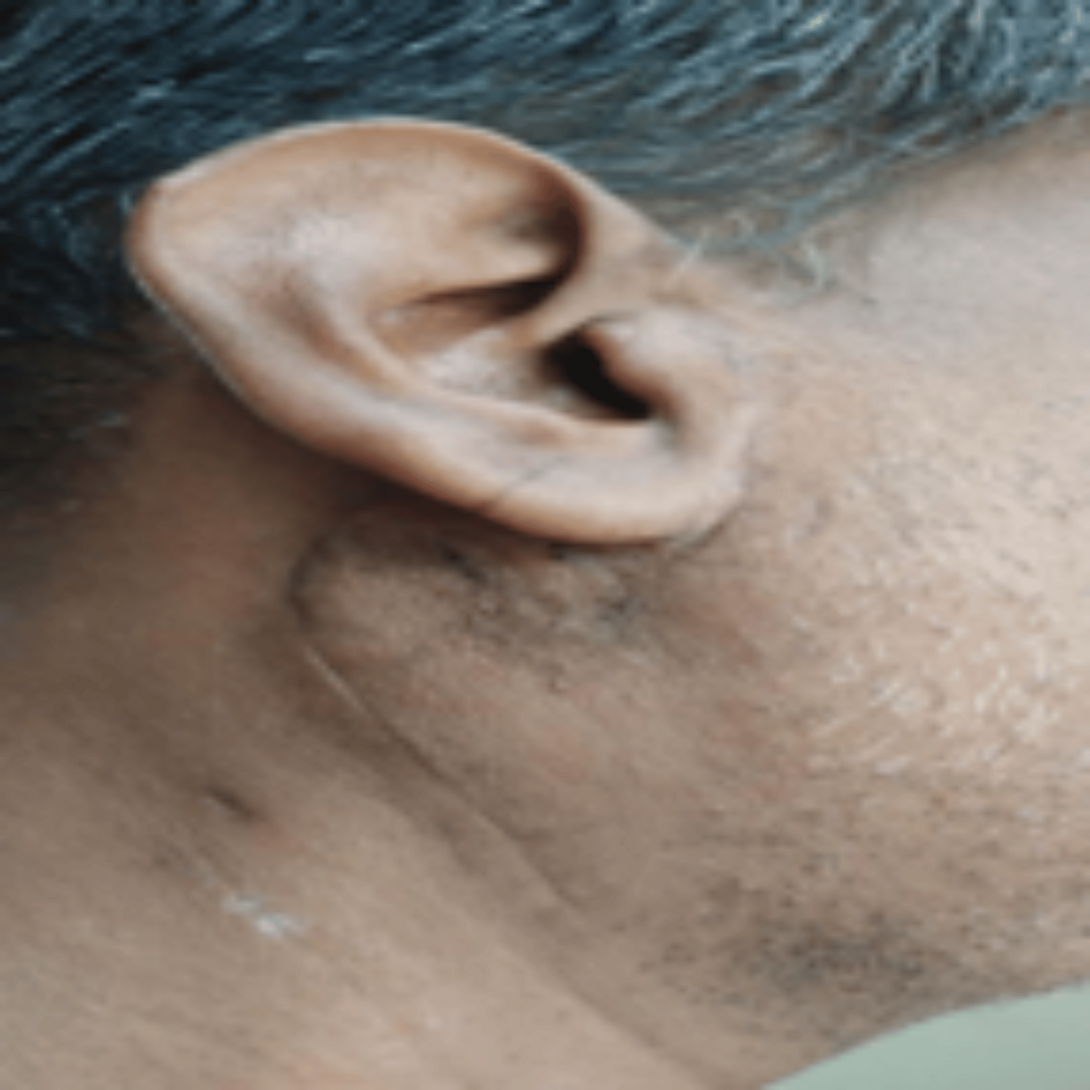
Postoperative photograph revealing the surgical scar

The patient’s postoperative course was uneventful, as depicted in Figure 4. The patient presented no symptoms consistent with recurrence, and regular follow-up was anticipated, with a positive response to therapy noted at the most recent visit. As histological findings confirmed the oncocytoma as benign, no further treatment was deemed necessary.

## Discussion

This case demonstrates the diagnostic and therapeutic challenges associated with a parotid oncocytoma, a rare and occasionally enigmatic benign tumor with clinical features potentially resembling those of other benign and low-grade malignant salivary gland neoplasms. Histopathological features included large polygonal cells with abundant eosinophilic cytoplasm, consistent with oncocytic characteristics. The findings align with a study conducted by Amir et al. (2022) [9], who identified oncocytoma cells exhibiting granular cytoplasm and benign growth patterns in a parotid tumor case.

Diagnosing parotid oncocytomas is notoriously difficult, as illustrated by various studies and case reports. For instance, a series of studies involving seven patients with parotid oncocytomas demonstrated comparable MRI features, including hypointense T1 signals and significant enhancement on contrast imaging. The present case closely corresponds to these findings, underscoring the utility of MRI in diagnostic results [3].

Korbi et al. (2019) noted that oncocytomas present a diagnostic challenge owing to their resemblance to other parotid tumors, with histological confirmation proving essential. Their patients’ successful outcomes following superficial parotidectomy support surgical excision as a first-line treatment, minimizing the risk of recurrence [4].

Research by Mamun et al. (2023) highlighted the histological ambiguity of oncocytomas, which can be misidentified as other benign tumors, such as pleomorphic adenomas or Warthin tumors. The authors emphasized the imperativeness of careful histological analysis to differentiate oncocytomas from other common parotid swellings. The current case reinforces the importance of multiple FNAC assessments, which did not yield the diagnosis until histological investigation postoperatively [2].

Histological investigations, as discussed by Kirkland et al. (2021), further elucidated the role of radionuclide imaging (e.g., single-photon emission computed tomography/computed tomography and sestamibi scintigraphy) in parotid oncocytomas. Although advanced imaging was not utilized in this patient, it may serve as a valuable diagnostic adjunct to differentiate benign oncocytomas from malignant neoplasms [7].

Sayad et al. (2021) characterized the histopathological features of oncocytomas - aligning with the findings in this case - as encapsulated lesions comprising mitochondrion-rich oncocytes. While these tumors are typically encapsulated and noninvasive, the authors advocated for surgical excision as the primary treatment and means of establishing a definitive diagnosis with a minimal risk of recurrence [1]. Imran et al. (2020) emphasized that while imaging results can often be nonspecific and overlap with other parotid tumors, they remain the gold standard for detecting oncocytomas [6]. This case corroborates their findings, as histological analysis of the excised tissue confirmed the diagnosis of an oncocytoma, highlighting the necessity of thorough microscopic examination [6]. The recurrence of parotid oncocytomas illustrates the significance of meticulous surgical management and histopathological correlation. Despite being benign, parotid oncocytomas can pose diagnostic challenges akin to those associated with other parotid gland tumors. Superficial parotidectomy proves effective as a therapeutic approach, yielding no postoperative recurrence and confirming the histological diagnosis of an oncocytoma.

## Conclusions

The initial diagnosis in this case was complicated by its unique clinical presentation, characterized by recurrent parotid swelling and abscess formation. Although MRI and FNAC provide valuable diagnostic insights, definitive diagnosis is exclusively achievable through histological analysis of excised tissue. Histopathology confirmed the presence of an oncocytoma, revealing a well-defined lesion comprising oncocytic cells against a backdrop of multifocal oncocytic hyperplasia. Consequently, surgical excision is paramount for establishing a definitive diagnosis and treatment. The successful management of this patient via superficial parotidectomy underscores the importance of surgical intervention, which effectively removes the lesion while preserving surrounding tissues and mitigating the likelihood of recurrence. The patient’s favorable outcome, characterized by the absence of recurrence and minimal postoperative morbidity, emphasizes the necessity of a comprehensive diagnostic process and precise surgical technique in managing benign oncocytomas. This case provides valuable insights into the relatively obscure nature of parotid oncocytomas and underlines the critical importance of early and accurate diagnosis to optimize patient outcomes.

## References

[1] Sayad Z, Dani B, Elazzouzi R, Benazzou S, Boulaadas M (2021). A rare tumor of the parotid gland: a case report and review of the literature. Int J Adv Res.

[2] Mamun MA, Islam MR, Majumder RA (2023). Oncocytoma: a mystifying parotid gland tumour. Mugda Medical College Journal.

[3] Lv K, Cao X, Geng D, Zhang J (2021). Imaging features of parotid gland oncocytoma: a case series study. Gland Surg.

[4] Korbi AE, Jellali S, Njima M (2019). Parotid gland oncocytoma: a rare case and literature review. J Med Cases.

[5] Corvino A, Caruso M, Varelli C (2021). Diagnostic imaging of parotid gland oncocytoma: a pictorial review with emphasis on ultrasound assessment. J Ultrasound.

[6] Imran S, Allen A, Shokouh-Amiri M, Garzon S, Saran N (2020). Parotid oncocytoma: CT and pathologic correlation of a rare benign parotid tumor. Radiol Case Rep.

[7] Kirkland JW, Zhao JM, McWhorter NE, Banks KP (2021). Parotid oncocytoma on 99mTc-sestamibi scintigraphy and SPECT/CT. Clin Nucl Med.

[8] Hammami B, Thabet W, Kallel R, Boudawara T, Mnejja M, Charfeddine I (2021). Bilateral multifocal nodular oncocytic hyperplasia of the parotid gland: a rare entity. Pathologica.

[9] Amir M, Aimon S, Latif S, Farooqui F (2022). Parotid gland oncocytoma: a case report. J Islam Int Med Coll.

